# Use of fixed calcium to phosphorus ratios in experimental diets may create bias in phytase efficacy responses in swine

**DOI:** 10.1093/tas/txac124

**Published:** 2022-08-29

**Authors:** Hengxiao Zhai, Jon R Bergstrom, Jingcheng Zhang, Wei Dong, Zhenzhen Wang, Kostas Stamatopoulos, Aaron J Cowieson

**Affiliations:** DSM (China) Animal Nutrition Research Center, Bazhou 065799, China; DSM Nutritional Products, Parsippany, NJ 07054, USA; DSM (China) Animal Nutrition Research Center, Bazhou 065799, China; DSM (China) Animal Nutrition Research Center, Bazhou 065799, China; DSM (China) Animal Nutrition Research Center, Bazhou 065799, China; DSM Nutritional Products, Kaiseraugst 4303, Switzerland; DSM Nutritional Products, Kaiseraugst 4303, Switzerland

**Keywords:** available, bone, calcium, digestibility, phosphorus, phytase

## Abstract

The objective of this study was to investigate the effects of two dietary total Ca/P ratios on available P release by phytase, measured using growth performance and bone mineralization with 528 barrows and gilts according to a randomized complete block design. Three were 11 diets in a factorial of 2 by 4 plus 3, including 3 reference diets consisting of 0.25% (control), 0.70%, or 1.15% monocalcium phosphate (MCP) and 8 diets from combining 4 phytase doses (500, 1,000, 2,000, and 3,000 FYT/kg) with 0.25% MCP and 2 dietary Ca/P ratios (1.05 and 1.20). Each diet was fed to 6 pens of 8 pigs. All diets contained 3 g/kg TiO_2_, and fecal samples were collected from each pen on d 13–15 of trial. At the end of trial, one pig per pen was sacrificed to collect a tibia and urine in the bladder. The results showed that MCP improved growth performance linearly (*P* < 0.01), whereas both a linear and quadratic response was observed with the addition of phytase. The MCP increased the percent bone ash and weights of bone ash, Ca, and P linearly (*P* < 0.01). At both Ca/P ratios, increasing supplementation of phytase increased the percent bone ash and weights of bone ash, Ca, and P both linearly and quadratically (*P* < 0.05). Both MCP and phytase significantly increased digestibility of Ca and P as well as digestible Ca and P in diets and reduced the digestible Ca/P ratio. The dietary Ca/P ratio of 1.20 resulted in poorer feed utilization efficiency, more digestible Ca, greater percent bone ash, Ca, and P and heavier weights of bone Ca and P than the ratio of 1.05 (*P* < 0.05). The ratio of 1.20 elicited numerically higher available P release values from phytase, with percent bone ash and bone P weight as the response variables, but significantly lower values with gain:feed. The urinary concentration of Ca increased linearly (*P* < 0.01) with increasing digestible Ca/P ratios whilst urinary concentration of P decreased quadratically (*P* < 0.01). In conclusion, fixing the same total Ca/total P ratio in diets supplemented with increasing phytase dosing created an imbalance of digestible Ca and P, which could have an adverse effect on bone mineralization and thus compromise the phytase efficacy relative to mineral P.

## INTRODUCTION

Phytase efficacy, in terms of available P release, was calculated from a standard response curve established by plotting a sensitive response variable such as bone ash against the level/intake of mineral P with an assumed P bioavailability of 100% (Cromwell et al., 1993; Kornegay and Qian, 1996; Wensley et al., 2020a). An underlying assumption is that dietary P supply, when it’s not exceeding the requirement and Ca is not limiting, is a good predictor for bone mineralization. Increasing dietary Ca also increases bone mineralization until its maximum, as long as P and Ca are balanced (Létourneau-Montminy et al., 2012). Either excessive or inadequate supply of Ca in a diet has negative impacts on bone mineralization. Lagos et al (2021) summarized the standardized total tract digestible (STTD) Ca/STTD P ratios to be 1.70:1, 1.80:1, 2:00:1, and 2.30:1 to maximize bone ash for 11–22, 20–50, 50–85, and 100–130 kg pigs, respectively, when P was at adequate. Theoretically, this digestible system can guarantee an optimal balance between Ca and P in diets for pigs. In the field, the Ca and P homeostasis is worth monitoring to maintain an optimal balance between Ca and P and to minimize P footprint on the environment. The urinary concentrations of Ca and P should be explored as potential markers because an oversupply of Ca or P was shown to trigger an increase in their excretion through urine (Rodehutscord et al., 1999; Stein et al., 2011).

In pig studies to measure available P release by phytase, the dietary Ca levels (Cromwell et al., 1993) or ratios of total Ca/P were fixed. For example, a total Ca/P ratio of 2:1 was used by Kornegay and Qian (1996), a ratio of 1.10:1 (0.97 to 1.10 on analysis) by Wensley et al. (2020a), and a ratio between 1.2 and 1.3 (1.31 to 1.39 on analysis) by Dersjant-Li et al. (2020). The total Ca and P do not reflect their different digestibility among feed ingredients, which implies that the same total Ca/P ratios in diets of different ingredient composition might correspond to disparate digestible Ca/P ratios. In addition, the contribution of digestible Ca by phytase has not been described precisely, and the issue of dissimilar digestible Ca/P ratios could be even worse between the reference diets and phytase diets in studies to determine the available P release for phytase.

The aim of the current study, therefore, was to investigate the effects of two total Ca/P ratios (1.05 and 1.20) on the growth performance, digestibility of Ca and P, bone mineralization, and urinary concentrations of Ca and P in nursery pigs supplemented with different doses of a novel phytase. We hypothesized that the bone mineralization will be greater when there is slightly more Ca in the phytase-supplemented diets and, therefore, the measured available P release by phytase as benchmarked to monocalcium phosphate (MCP) will be elevated.

## MATERIALS AND METHODS

This study was conducted at DSM (China) Animal Nutrition Research Center Co. Ltd. (Bazhou, P. R. China), and its protocol was approved by the Animal Welfare Committee of DSM (China) Animal Nutrition Research Center (AWCCAN). The guidelines in European Union council directive 2010/63/EU for animal experiments were followed in this study.

### Animals and Facilities

Five hundred and twenty-eight barrows and gilts (PIC L1050 × L337; initial body weight 7.4 ± 0.9 kg [mean ± standard deviation]) were used. The pigs were weaned at an average age of 21 d and transferred to a nursery facility for an adaptation period of 7 d using a common starter diet. The nursery facility was equipped with 80 pens (space/pen = 3.0 × 1.8 m^2^). Each pen had a plastic-coated wire floor and was equipped with two water nipples and one stainless-steel feeder. After the adaptation period, the pigs were individually weighed and allotted into 66 pens based on their initial BW and gender (four barrows and four gilts per pen). Each pen of pigs was assigned to 1 of 11 dietary treatments in a Randomized Complete Block Design, resulting in 6 replicate pens per dietary treatments. The experimental diets were fed for 21 d, with feed and water supplied ad libitum. At the end of trial, the pigs weighed 17.1 ± 2.1 kg.

Room temperature and ventilation were controlled by a computer system to provide an optimal environment. The room temperature was 27 °C at the start and gradually reduced to 23 °C at the end. The relative humidity was recorded to range from 40% to 60%.

### Experimental Diets

The ingredient and nutrient composition of the basal diet are presented in Table 1. There were 11 experimental diets. Three diets were established by including 0.25% (control), 0.60%, or 0.95% MCP to establish the standard response curves. Four more diets were created by including the phytase at 500, 1,000, 2,000, or 3,000 FYT/kg feed in the control diet. The test phytase (HiPhorius, DSM Nutritional Products, Switzerland) was encoded by a 6-phytase gene from *Citrobacter braakii* and expressed from a strain of *Aspergillus oryzae*. In these seven diets, the formulated ratio of total Ca to total P was maintained at 1.05 by adjusting the inclusion level of limestone. An additional four diets also included 500, 1,000, 2,000, or 3,000 FYT/kg phytase but with a formulated total Ca/P ratio of 1.20 using adjusted inclusion levels of limestone. Titanium dioxide was included at 3 g/kg feed as an indigestible marker to enable the measurement of apparent total tract digestibility (ATTD) of Ca and P in all diets. The net energy and nutrients, other than Ca and P, were above the recommendations in NRC (2012) for all the experimental diets. All diets were pelleted with a conditioning temperature at 75 °C.

**Table 1.** Ingredient and nutrient composition of the basal diet (g/kg of feed, as-is basis)

Items	Basal diet
Ingredients	
Corn	590.9
Soybean meal	340.5
Soybean oil	25.0
NaCl	4.5
NaHCO_3_	1.5
L-Lys·HCl	4.5
DL-Met	1.2
L-Thr	1.5
L-Val	1.0
Limestone	6.8
Monocalcium phosphate	2.5
Vitamin-mineral premix^1^	5.0
Rice hull	9.1
Benzoic acid	3.0
TiO_2_	3.0
Total	1,000.0
Calculated nutrients and energy	
Net energy, kcal/kg	2,499
Metabolizable energy, kcal/kg	3,372
Crude protein	21.8
Total Ca	4.5
Total P	4.2
Phytate P	2.5
Standardized total tract digestible P	2.0
Standardized ileal digestible	
Lys	12.9
Met	3.9
Thr	7.7
Trp	2.1
Val	8.3

Premix supplied per kilogram of diet: vitamin A, 9,750 IU; vitamin D_3_, 3,000 IU; vitamin E, 63 mg; vitamin K_3_, 3.0 mg; vitamin B_1_, 3.0 mg; vitamin B_2_, 9.6 mg; vitamin B_6_, 4.5 mg; vitamin B_12_, 36 μg; D-biotin, 240 ug; D-calcium pantothenate, 30 mg; folic acid, 1.8 mg; niacin, 36 mg; Cu (tribasic copper chloride), 190 mg; I (potassium iodate), 0.6 mg; Fe (ferrous sulfate), 120 mg; Mn (manganese sulfate), 60 mg; Zn (zinc sulfate), 120 mg; Se (sodium selenite), 450 μg; choline (choline chloride), 300 mg; and Ca (calcium carbonate) 0.6 g.

### Measurement and Sampling

The pigs were individually weighed on day 0 and 21 of trial to obtain the total weight for each pen of pigs, and the feed consumption per pen was recorded during the trial to calculate average daily gain (ADG), average daily feed intake (ADFI), and gain:feed.

Fresh and clean fecal samples were grabbed from each pen on day 13 to 15 of trial. All the pens were cleaned, and existing feces removed before collection on each collection day. A total of approximately 500 g of fresh feces was collected per day per pen. All the fecal samples collected from each pen during the 3-d collection period were pooled and mixed to homogeneity with a hand-held blade mixer (TD-110, RuiBao Hardware Co. Ltd., Dongguan, P. R. China). A subsample of around 400 g for each pen was collected and stored at −20 °C until further processing.

The right tibia and urine from the bladder (if abundant at sampling) were collected from the pig in each pen with body weight closest to the average body weight per pen on d 21 of trial. The tibias were processed by referring to the non-defatting bone processing procedures described by Wensley et al. (2020b). In short, the bones were autoclaved at 120 °C for 30 min to facilitate the removal of muscular tissues and cartilaginous caps. The cleaned bones were left at room temperature for 1 d and then oven-dried at 105 °C for 7 d. In the end, the dried tibias were incinerated in a muffler oven for 72 h at 600 °C. All the samples were stored at −20 °C before processing.

### Chemical Analyses

The fecal samples were oven-dried to a constant weight and ground to pass through a 0.5-mm screen before analysis. The dietary and fecal samples were dried at 105 °C in an oven for 4 h for dry matter determination (method 934.01; AOAC International, 2006). Titanium, Ca, and P were determined by Inductively Coupled Plasma-Optical Emission Spectrometry (ICP-OES; Optima TM 8000, PerkinElmer, Shelton, USA; method 985.01; AOAC International, 2006) after microwave digestion. Ten mL of each urine sample were dried at 60 °C before the microwave digestion. One phytase unit (FYT) was defined as the amount of enzyme that releases 1 µmol of inorganic phosphate from 50 mM phytate per minute at 37 °C and pH 5.5. These analyses were performed in duplicate, except that phytase activity in the feed samples was determined from three replicates.

### Calculations and Statistical Analyses

The experiment was a randomized complete block design. Each pen or pig was an experimental unit.

Digestibility of Ca and P was calculated using the following equation:


$$\begin{array}{l} D=\;\left[1-\left(T_{i}/T_{o}\right)\times \left(M_{o}/M_{i}\right)\right]\times 100; \end{array}$$

where *D* is the digestibility of Ca or P (%); *T*_i_ and *T*_o_ are the titanium concentrations in diet and feces, respectively (% of *DM*); *M*_i_ and *M*_o_ are the concentrations of Ca or P in diet and feces (% of *DM*), respectively. The digestible Ca and P were calculated by multiplying the concentrations of Ca and P in feed (g/kg feed) by their corresponding digestibility coefficients.

Available P release by phytase was calculated by referring to the method described by Wensley et al. (2020a). The standard response curves were established by regressing ADG against dietary P intake (g/d) and regressing gain:feed, bone ash percent, and bone P weight against dietary P concentration (%). The feed intake was incorporated for the response curve of ADG to achieve a greater fitting to the results. Using the standard response equations, the dietary P concentration equivalence was solved for each dose of phytase. These equivalent values were corrected for the contribution by the control diet to generate the available P release values for phytase.

The data were analyzed using the GLM procedure of SAS (SAS Inst. Inc., Cary, NC) with the model including the dietary treatment as a fixed effect, replicate as a random effect, and an error term. Polynomial orthogonal contrasts were constructed to test the linear and quadratic effects of supplementation of MCP and phytase, the effect of dietary Ca/P ratio among phytase diets, and the interaction between phytase and the Ca/P ratio. The least-square means were presented, and the significance was defined at *P* < 0.05.

## RESULTS

### Experimental Diets and the Analyses

The total P in diets with 0.25% MCP was analyzed to be 0.45%–0.47%, and incremental increases of 0.08%–0.09% P agree with the increasing dietary inclusion of MCP from 0.25% to 0.60% to 0.95% (Table 2). The analyzed total Ca/P ratios ranged from 1.02 to 1.07 for the target of 1.05 and from 1.19 to 1.20 for 1.20. The analyzed marker concentrations ranged from 97% to 101% of the formulated value. The analyzed phytase activities were within ± 15% of the intended doses.

**Table 2.** Analyzed nutrients of the dietary treatments (as-is basis, %)^1^

Diet	Ca/P ratio	Ca, %	P, %	Ca/P ratio	Phytase, U/kg
MCP, %					
0.25	1.05	0.50	0.47	1.07	0
0.60	1.05	0.57	0.55	1.05	0
0.95	1.05	0.67	0.64	1.05	0
Phytase, U/kg					
500	1.05	0.47	0.46	1.02	511
1,000	1.05	0.50	0.47	1.06	1,009
2,000	1.05	0.49	0.46	1.07	2,110
3,000	1.05	0.48	0.47	1.03	3,333
500	1.20	0.54	0.45	1.20	573
1,000	1.20	0.54	0.45	1.19	1,112
2,000	1.20	0.55	0.46	1.19	2,292
3,000	1.20	0.55	0.46	1.19	3,240

Phytase activity was analyzed in three replicates and the others in duplicate.

### Growth Performance and Bone Mineralization

The interaction between phytase and the Ca/P ratio was not significant for growth performance. Increasing supplementation of MCP linearly improved (*P* < 0.01) final BW, ADG, ADFI and gain:feed, whereas both linear and quadratic responses, were observed with the addition of phytase (*P* < 0.01; Table 3). Reducing the Ca/P ratio from 1.20 to 1.05 significantly increased gain:feed from 766 to 778 g/kg.

**Table 3.** Growth performance of the pigs supplemented with monocalcium phosphate (MCP) or phytase^1^

Diet		IBW^2^, kg	FBW^2^, kg	ADG^2^, g/d	ADFI^2^, g/d	Gain: feed, g/kg
MCP, %						
0.25		7.4	14.9	359	521	691
0.60		7.4	16.0	410	563	729
0.95		7.4	16.8	447	591	757
Phytase, U/kg						
500		7.4	16.9	451	602	749
1,000		7.4	17.6	484	628	771
2,000		7.4	17.7	490	627	782
3,000		7.4	17.7	490	624	787
Ca/P ratio						
1.05		7.4	17.5	480	618	778
1.20		7.4	17.4	477	623	766
	SD^3^	0.09	0.43	20	26	19
*P* value						
MCP	L^4^	0.28	<0.01	<0.01	<0.01	<0.01
	Q^4^	0.60	0.46	0.52	0.61	0.63
Phytase	L^5^	0.71	<0.01	<0.01	<0.01	<0.01
	Q^5^	0.40	<0.01	<0.01	<0.01	<0.01
Ca/P ratio^6^		0.68	0.64	0.59	0.55	<0.05

There were six replicates of eight pigs.

IBW, initial body weight; FBW, final body weight; ADG, average daily gain; ADFI, average daily feed intake.

SD, standard deviation.

Linear and quadratic effects of monocalcium phosphate.

Linear and quadratic effects of phytase.

The effect of Ca/P ratio compares phytase diets between the ratios of 1.05 and 1.20, and no significant interaction between phytase and the Ca/P ratio was observed.

There was no significant interaction between phytase and the Ca/P ratio for bone mineralization. Incremental increases in MCP linearly increased (*P* < 0.01) percent bone ash and weights of bone ash, Ca, and P (Table 4). Increasing the addition of phytase increased the percent bone ash and weights of bone ash, Ca and P, both linearly and quadratically (*P* < 0.05). The bone Ca/P ratio decreased linearly (*P* < 0.01) with increasing supplementation of MCP and quadratically (*P* < 0.01) with supplemental phytase. The dietary Ca/P ratio of 1.20 resulted in greater percent bone ash Ca and P, a heavier weight of bone Ca, and a higher Ca/P ratio in bone than the dietary Ca/P ratio of 1.05 (*P* < 0.05).

**Table 4.** Bone mineralization of the pigs supplemented with monocalcium phosphate (MCP) or phytase^1^

Diet		Bone ash, %	Bone ash Ca, %	BoneashP, %	Bone ash, g	Bone Ca, g	Bone P, g	Bone Ca/P ratio
MCP, %								
0.25		43.7	33.8	16.9	3.18	1.07	0.53	2.00
0.60		46.0	33.6	17.0	4.01	1.34	0.68	1.98
0.95		50.1	33.4	17.2	5.09	1.70	0.88	1.94
Phytase, U/kg								
500		48.0	33.9	17.3	4.51	1.51	0.78	1.96
1,000		48.2	34.0	17.6	4.87	1.64	0.85	1.92
2,000		50.6	34.4	17.6	5.28	1.80	0.93	1.94
3,000		50.2	34.6	17.7	5.36	1.84	0.94	1.95
Ca/P ratio								
1.05		49.0	33.6	17.4	4.95	1.65	0.86	1.93
1.20		49.5	34.8	17.8	5.06	1.75	0.90	1.96
	SD^2^	2.20	1.44	0.59	0.41	0.15	0.07	0.04
*P* value								
MCP	L^3^	<0.01	0.67	0.32	<0.01	<0.01	<0.01	<0.01
	Q^3^	0.41	0.98	0.70	0.53	0.61	0.45	0.46
Phytase	L^4^	<0.01	0.14	<0.01	<0.01	<0.01	<0.01	0.09
	Q^4^	<0.01	0.92	0.08	<0.01	<0.01	<0.01	<0.01
Ca/P ratio^5^		0.41	<0.01	0.03	0.36	0.03	0.06	0.04

There were six replicates of one pig.

SD, standard deviation.

Linear and quadratic effects of monocalcium phosphate.

Linear and quadratic effects of phytase.

The effect of Ca/P ratio compares phytase diets between the ratios of 1.05 and 1.20, and no significant interaction between phytase and the Ca/P ratio was observed.

### Digestibility of Ca and P, Digestible Ca and P in Experimental Diets, and Urinary Concentrations of Ca and P

The interaction between phytase and the Ca/P ratio was significant for digestibility of Ca and digestible Ca. This significant interaction was due to continued increase in digestibility of Ca and digestible Ca beyond the phytase dose of 1,000 FYT/kg at the Ca/P ratio of 1.20 in contrast to the plateaued response at the Ca/P ratio of 1.05. Increasing MCP or phytase in the diets, irrespective of the dietary Ca/P ratio, improved digestibility of P and digestible P, but reduced the digestible Ca/P ratio, both linearly and quadratically (*P* < 0.05; Table 5). Digestible Ca was linearly increased (*P* < 0.01) with increasing dietary inclusion of MCP. The high dietary Ca/P ratio significantly increased the digestible Ca/P ratio compared to the low dietary Ca/P ratio (1.28 vs 1.10).

**Table 5.** Apparent total tract digestibility (ATTD) of Ca and P in the experimental diets supplemented with monocalcium phosphate (MCP) or phytase^1^

Diet	Ca/P ratio	ATTD ofCa, %	ATTD ofP, %	Digestible Ca, g	Digestible P, g	Digestible Ca/P
MCP, %						
0.25	1.05	56.2	38.9	2.82	1.83	1.55
0.60	1.05	62.8	50.3	3.58	2.74	1.31
0.95	1.05	64.1	53.5	4.28	3.39	1.26
Phytase, U/kg						
500	1.05	71.3	62.6	3.33	2.88	1.16
1,000	1.05	77.9	72.0	3.86	3.37	1.15
2,000	1.05	75.7	75.9	3.70	3.48	1.06
3,000	1.05	80.1	81.9	3.86	3.82	1.01
500	1.20	73.7	65.4	3.96	2.94	1.35
1,000	1.20	76.4	71.2	4.15	3.23	1.28
2,000	1.20	80.3	78.0	4.41	3.58	1.23
3,000	1.20	84.8	80.7	4.68	3.73	1.25
	SEM^2^	0.94	0.65	0.05	0.03	0.02
*P* value						
MCP	L^3^	<0.01	< 0.01	< 0.01	< 0.01	< 0.01
	Q^3^	0.03	< 0.01	0.59	< 0.01	< 0.01
Phytase (1.05)	L^4^	< 0.01	< 0.01	< 0.01	< 0.01	< 0.01
	Q^4^	< 0.01	< 0.01	< 0.01	< 0.01	< 0.01
Phytase (1.20)	L^5^	< 0.01	< 0.01	< 0.01	< 0.01	< 0.01
	Q^5^	< 0.01	< 0.01	< 0.01	< 0.01	< 0.01
Ca/P ratio^6^		< 0.01	0.12	< 0.01	0.50	< 0.01

There were six replicate pens.

SEM, standard error of the mean.

Linear and quadratic effects of monocalcium phosphate.

Linear and quadratic effects of phytase at the Ca/P ratio of 1.05.

Linear and quadratic effects of phytase at the Ca/P ratio of 1.20.

The effect of Ca/P ratio compares phytase diets between the ratios of 1.05 and 1.20, and a significant interaction effect between phytase and the Ca/P ratio was observed for digestibility of Ca and digestible Ca.

**Figure 1.**
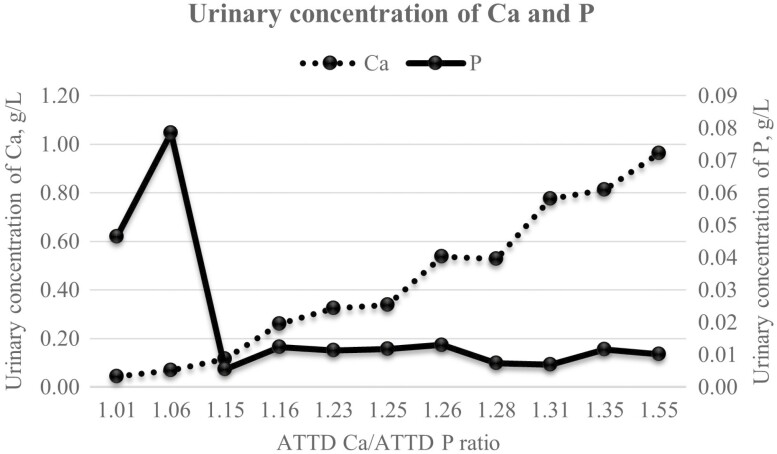
Urinary concentrations of Ca (linear effect: *P* < 0.01) and P (quadratic effect: *P* < 0.01) with increasing ratios of apparent total tract digestible (ATTD) Ca over ATTD P.

The urinary concentration of Ca increased linearly (*P* < 0.01) with increasing digestible Ca/P ratio whilst urinary concentration of P increased quadratically (*P* < 0.01; Figure 1).

### Standard Response Curves

The standard regression equations were Y=76.6×X+168.8 (*r*^2^ = 0.94), Y=396.3×X+507.8 (*r*^2^ = 0.62), Y=39.2×X+25.1 (r^2^ = 0.61), and Y=2.08×X+0.45 (*r*^2^ = 0.78), respectively, for ADG, gain:feed, percent bone ash and bone P weight, respectively.

### The Comparison of Available P Release by Phytase

The interaction between phytase and the Ca/P ratio was not significant (Table 6). Using ADG and bone P weight as response variables, available P release increased both linearly and quadratically (*P* < 0.05) with increasing phytase dose, whereas a linear (*P* < 0.01) increase was observed based on gain: feed and percent bone ash. The Ca/P ratio of 1.05 tended to increase available P release based on gain: feed (0.221 vs 0.192) when compared to the ratio of 1.20, but the opposite tendency (0.167 vs 0.186) was shown by bone P weight (*P* < 0.10).

**Table 6.** Comparison of available P release values based on different response variables, g/kg feed^1^

Response variable	1.05 Ca/P				1.20 Ca/P				SD^2^	P value		
	Phytase, FYT/kg feed				Phytase, FYT/kg feed					Phytase		Ca/P ratio^4^
	500	1,000	2,000	3,000	500	1,000	2,000	3,000		L^3^	Q^3^	
ADG^5^, g/d	0.158	0.210	0.199	0.221	0.139	0.180	0.216	0.204	0.03	< 0.01	< 0.01	0.12
Gain:feed, g/kg	0.177	0.234	0.212	0.262	0.119	0.176	0.249	0.226	0.05	< 0.01	0.06	0.05
Bone ash, %	0.117	0.110	0.192	0.182	0.132	0.149	0.193	0.180	0.05	< 0.01	0.18	0.39
Bone P, g	0.125	0.148	0.198	0.197	0.131	0.184	0.207	0.221	0.04	< 0.01	0.04	0.09

There were six replicates.

SD, standard deviation.

Linear and quadratic effects of phytase.

The effect of Ca/P ratio compares between the ratios of 1.05 and 1.20, and no significant interaction between phytase and the Ca/P ratio was observed.

ADG, average daily gain.

## DISCUSSION

### The Diets and Animals

In general, the analyzed dietary concentrations of Ca and P were slightly higher than the formulated values, but the agreement between the calculated and formulated ratios lent credence to the experimental diets to test the hypothesis of this study. Moreover, the analyzed phytase activities were consistent with their intended values. The stepwise increases in both analyzed Ca and P agreed with the incremental additions of MCP, which are prerequisites for establishing the standard response curves. The animals were in good health throughout the trial. The culling rate was only 1.0%.

### Phytase Efficacy

The measured growth performance, digestibility of Ca and P, and bone mineralization in this study followed a classical, curvilinear trend in response to the increasing levels of phytase, which attests to the basic function of phytase to liberate Ca and P. The improvement in body weight gain with added phytase was attributed to an increase in both feed intake and feed utilization efficiency. A deficiency of P can cause a poor appetite in pigs (Jongbloed, 1987), so the addition of phytase to a P-deficient diet should restore appetite by providing bioavailable P. The improvement in feed utilization efficiency is supported by the close relationship between whole-body P mass and whole-body N mass (NRC, 2012). Soft tissue development is dependent mainly on P even though bone mineralization requires both Ca and P (Létourneau-Montminy et al., 2012). Moreover, the preference of most exogenous phytases for breaking down *myo*-inositol hexakisphosphate (IP_6_) and IP_5_ (Wyss et al., 1999; Pontoppidan et al., 2012) means there will be less residual phytate exerting antinutritional effects because IP_6_ and IP_5_ are more capable of binding protein and minerals than other IP esters (Yu et al., 2012). In addition, *myo*-inositol released from complete destruction of phytate at high phytase doses might provide some extra-phosphoric effects (Schmeisser et al., 2017; Lu et al., 2019).

There were some interesting observations about bone mineralization. First, phytase increased percentage and weight of bone ash irrespective of the dietary Ca/P ratio, but a further improvement in the percentage of Ca and P in bone ash was observed only at the dietary Ca/P ratio of 1.20. This implies that weights of Ca and P in bone ash are more sensitive than bone ash weight. Second, more available Ca at the dietary Ca/P ratio of 1.20 than at 1.05 led to a higher Ca/P ratio in the bone. It appears that bone has a certain degree of plasticity in terms of the ratio of Ca/P in it. However, it is generally considered that the ratio of Ca/P in bone is about 2.1:1 which is tightly regulated due to the finite chemical structure of hydroxyapatite of bone (Cromwell, 2005). Thirdly, our Ca/P ratios in bone are much lower than the ratios reported by González-Vega et al. (2016) and Lagos et al. (2019), which indicates that there is still space for even higher dietary Ca/P ratios to elicit greater bone development.

### The Effects of Dietary Total Ca to Total P Ratio

In the current study, increasing dietary Ca/P ratio from 1.05 to 1.20 impaired feed utilization efficiency, but increased digestibility of Ca as well as the amount of digestible Ca, and consequently increased the amounts of Ca and P deposited in bone. These results indicate that slightly more Ca in phytase-supplemented diets could result in more Ca digested and absorbed without diminishing the efficacy of phytase in terms of P release and greater bone mineralization could be realized. This agrees with the finding by Létourneau-Montminy et al. (2010) that reducing the dietary Ca/P ratio from 1.9 to 1.3 in a practical diet containing 0.56% P did not improve the efficiency of phytase in releasing P, but impaired bone mineralization in weanling pigs. Increasing dietary Ca will increase the amount of retained P as long as Ca and P are balanced or until bone mineralization reaches a plateau (Létourneau-Montminy et al., 2012). The impairment of gain:feed by the high Ca/P ratio agrees with the finding by Johnston et al. (2004) that the reduction in dietary Ca and P was just as effective as dietary phytase addition in increasing the digestibility of nutrients. The antinutritional effects of phytate could be aggravated by more Ca present in diet. It is well known that phytate negatively affects amino acids availability (Pontoppidan et al., 2007), starch digestion (Thompson, 1987), and fat utilization (Govers and Van der Meer, 1993), and is associated with greater endogenous losses of amino acids and minerals (Cowieson et al., 2009). The adverse effects of excess Ca could be mitigated by high doses of phytase as indicated by the significant interaction between phytase and the Ca/P ratio on digestible Ca.

These results with different dietary Ca/P ratios in the current study should be interpreted with prudence because we investigated only a narrow range of dietary Ca/P ratios corresponding to a difference in Ca level of only 0.05%. When the Ca/P ratio was examined in a wider range, different conclusions could be made. Qian et al. (1996) investigated three total Ca/P ratios of 1.2:1, 1.6:1, and 2.0:1 in diets supplemented with 700 or 1,050 U phytase/kg and reported adverse effects of wide Ca/P ratios on growth performance, bone characteristics, and P digestibility in weanling pigs. In growing-finishing pigs fed diets supplemented with 500 phytase U/kg, lowering the dietary Ca/P ratio from 1.5:1 to 1.3:1 to 1.0:1 improved growth performance and bone mineralization (Liu et al., 1998). In nursery pigs, a linear decrease in growth performance and bone mineral content was observed when the Ca/available P ratio increased from 1.25 to 2.75 in diets with 250 U/kg phytase added (Becker et al., 2020). These adverse effects associated with the wide Ca/P ratios could be due to a reduction in phytase efficacy (Qian et al., 1996), the formation of insoluble Ca-P complexes in the gastrointestinal tract (Stein et al., 2011), and the antinutritional effects of residual phytate due to low phytase dosing coupled with excess Ca. The extra supply of Ca can be expelled through urine, but has a negative impact on P digestibility (Stein et al., 2011). In the current study, a slight increase in Ca supply didn’t depress P digestibility and the improved digestible Ca/P ratio happened to be conducive to bone mineralization.

### Measuring Available P Release of Phytase

A fixed dietary Ca/P ratio was usually used to determine available P release from phytase. A ratio of 2:1 was used by Kornegay and Qian (1996), a ratio of 1.10:1 (0.97 to 1.10 on analysis) by Wensley et al. (2020a), and a ratio between 1.2 and 1.3 (1.31 to 1.39 on analysis) by Dersjant-Li et al. (2020). The total Ca and P do not reflect the different digestibility among feed ingredients, and the same total Ca/P ratios might result in different digestible Ca/P ratios. In the current study, the digestible Ca/P ratio decreased with increasing supplementation of either MCP or phytase, and the digestible Ca/P ratios were more closely correlated with the systemic homeostasis of Ca and P as indicated by urinary concentrations of Ca and P. Of note, only at the total Ca/P ratio of 1.20 did the digestible Ca/P ratios in the diets with phytase remained similar to the ratios in the reference diets with a total Ca/P ratio of 1.05. It appears that fixing the total Ca/P ratios in diets to compare MCP and phytase is not “fair” for phytase.

In general, the available P release values were higher at the total Ca/P ratio of 1.05 than at 1.20 when ADG and gain:feed were used as response variables, whereas the opposite was noted when using percent bone ash and bone P weight. This generality reflects the different effects of Ca/P ratios on growth performance and bone mineralization as discussed above. It is apparent that Ca plays a very important role in defining the P release values of phytase. The P release values of phytase should relate to the dietary supply of Ca, which depends on the production goals: growth performance *vs* bone development. More research is warranted to understand and address the plausible dilemma for nutritionists that different dietary levels of Ca are required for optimal growth performance and bone development. Using greater levels of phytase seems to be one possible solution. Separating feeding of Ca did not appear to be a valid solution (Pointillart and Guéguen, 1993). Different Ca sources should be investigated and compared in terms of their dissolution in gut and absorbability.

### The Homeostasis of Ca and P as Indicated by Urinary Concentrations of Ca and P

The urinary concentration of Ca increased with increasing ATTD Ca/P ratio, which agrees with the results by Stein et al. (2011) that increasing Ca supply from 55 to 173% of the requirement increased urinary excretion of Ca. Calcium homeostasis is mainly regulated in kidney rather than in gut in pigs (Stein et al., 2011). On the contrary, P homeostasis can be regulated at both renal and intestinal levels (Rodehutscord et al., 1999). The urinary concentration of P increased strikingly at the ratios of 1.01 and 1.06 when compared to the ratio of 1.15 or above, indicating the excretion of excess P through urine when there was a deficiency of Ca in relation to P. Pointillart and Fontaine (1983) also found more digestible P lost in urine because of a lack of Ca in the diet. The combination of concentrations of Ca and P in urine can be used as a powerful indicator for the balance of Ca and P supply for pigs. More research should be conducted to link the urinary concentrations of Ca and P with different degrees of bone mineralization. This relationship, if coupled with a real-time portable device to measure urinary Ca and P concentrations, can be a useful tool to gauge the balance of Ca and P in diet for pigs.

## CONCLUSION

In this study, increasing the dietary Ca/P ratio from 1.05 to 1.20 in diets supplemented with 500 to 3,000 FYT/kg phytase impaired feed utilization efficiency, but increased the digestible Ca in diet and the deposition of Ca and P in bone. This led to a tendency to elicit higher available P release values from phytase at the Ca/P ratio of 1.20 than at 1.05 when using bone P weight as the response variable. Therefore, fixing the same total Ca/total P ratio in diets supplemented with increasing phytase dosing created an imbalance of digestible Ca and P, which could have an adverse effect on bone mineralization and should be corrected with the appropriate addition of Ca. More research is warranted to precisely quantify the contribution of digestible Ca and P by phytase under different dietary and animal conditions, and this will enable a more precise supply of adequate Ca and P for nutrition.
